# Characterization of the NRPS operon homolog for surfactin A and surfactin C synthesis in *Bacillus* spp.

**DOI:** 10.1007/s00203-025-04341-z

**Published:** 2025-05-29

**Authors:** Kojiro Ito, Mana Adachi, Minenosuke Matsutani, Ryota Kataoka, Gen Enomoto, Akinobu Kajikawa, Kenji Yokota

**Affiliations:** 1 Department of Agricultural Chemistry, Tokyo University of Agriculture, Tokyo, Japan; 2 NODAI Genome Research Center, Tokyo University of Agriculture, Tokyo, Japan; 3 Present address: Department of Food, Aroma and Cosmetic Chemistry, Faculty of Bioindustry, Tokyo University of Agriculture, Hokkaido, Japan; 4 Faculty of Life and Environmental Sciences, University of Yamanashi, Yamanashi, Japan

**Keywords:** *Bacillus*, Cyclic lipopeptide, Surfactin, Non-ribosomal peptide synthesis

## Abstract

Surfactin is a cyclic lipopeptide produced by *Bacillus* spp., consisting of a β-hydroxy fatty acid and a heptapeptide synthesized by non-ribosomal peptide synthetases. Surfactin congeners (A, B, and C) differ in amino acid substitutions, with Leu7 in surfactin A replaced by Val in B and Ile in C. Our LC–MS analysis revealed that the elution profiles of surfactin-producing strains could be classified into two distinct patterns under identical culture conditions, corresponding to surfactin A and C production. This suggests that endogenous factors influence surfactin production. Therefore, we aimed to identify the genetic factor that regulates surfactin congener production. The *srfA* operon for surfactin A biosynthesis in *B. subtilis*, composed of four open reading frames (ORFs), is *srfAABCD*. Comparative genomic analysis between the *B. subtilis* JCM 1465 *srfA* operon and the TUA12 surfactin biosynthesis genes examined in this study revealed that the operon responsible for surfactin A biosynthesis is distinct, exhibiting 68.7%, 69.2%, 84.7%, and 67.4% homology with the four ORFs, respectively. Similarly, the operon for Ptrs2 surfactin C biosynthesis showed 68.7%, 69.2%, 64.4%, and 67.1% homology. These differences indicate that the identified surfactin A and C biosynthetic operons are novel genetic variants. Further analysis identified the adenylation domain responsible for selecting Ile7 in surfactin C via domain substitution in a surfactin A-producing strain. Average nucleotide identity analysis showed that the surfactin A and C operons were found in *B. velezensis* and *B. amyloliquefaciens*, respectively. Our findings suggest that surfactin congener production is species-dependent, with the *srf* operon specifically distributed in *Bacillus* spp.

**Supplementary Information:**

The online version contains supplementary material available at 10.1007/s00203-025-04341-z.

## Introduction

**Fig. 1:**
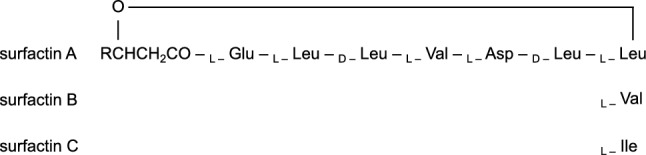
Chemical structure of surfactin congeners. R: C_11_-C_14_ alkyl chain

Cyclic lipopeptides (cLPs), derived from *Bacillus* spp., are secondary metabolites and biologically active compounds. Surfactins, consisting of a β-hydroxy fatty acid and heptapeptide moiety, are a type of cLPs in *Bacillus* species. Surfactin congeners (surfactins A, B, and C) vary in the primary structure of their heptapeptide moiety (Soberón-Chávez 2011) (Fig. 1).

The β-hydroxy fatty acid moiety in surfactin is biosynthesized by type II fatty acid synthesis (FAS) which conserved in bacteria (Diomandé et al. 2015). The surfactin congeners of surfactin in fatty acid moiety consist of the length of acyl chain and structural isomers of normal, iso and anteiso of fatty acids, and these structural isomers resulted by precursors in FAS. Acetyl-CoA or propionyl-CoA are used for normal acyl chain, whereas L-valine or L-leucine used for iso, and L-isoleucine for anteiso. The elongation of acyl chain is carried out by FabF, FabG, FabZ and FabI/FabL using acyl-ACP and malonyl-ACP as substrates (Hu et al. 2019), and the resulted acyl-CoA is incorporated into the NRPS complex by acyl-CoA ligase LcfA and LcfB (Kraas et al. 2010).

**Fig. 2:**
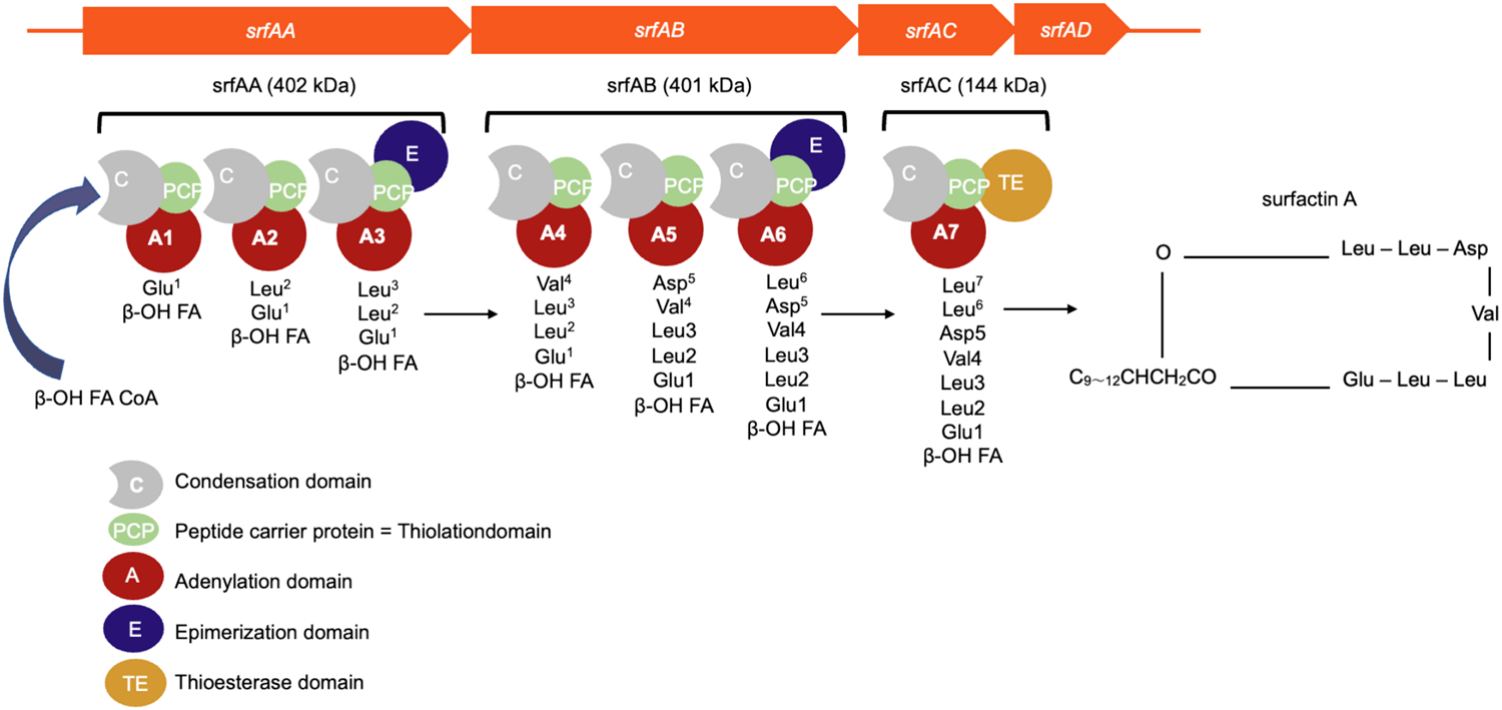
Surfactin biosynthetic gene cluster

Biosynthesis of the heptapeptide moiety of surfactin is performed by non-ribosomal peptide synthetases (NRPSs) (Nakano et al. 1991; Sieber and Marahiel 2005) (Fig. 2). Molecular mechanisms underlying peptide synthesis by NRPSs have been extensively studied. A module, consisting of an adenylation (A) domain for selecting and activating the amino acid as acyl adenylate, a thiolation (T)-domain, also called peptidyl-carrier protein (PCP), for the transport of activated intermediate amino acids as enzyme-bound thioesters, and a condensation (C) domain for catalyzing the formation of the peptide bond between acyl-S-PCP intermediates, is needed for the elongation of an amino acid. The NRPS operon for surfactin biosynthesis consists of four ORFs, *srfAA* (10.7 kbp), *B* (10.7 kbp), *C* (3.8 kbp), and *D* (0.7 kbp), and was identified as the *srfA* operon in *B. subtilis* JH 642 by Galli et al. (1994).

Some studies have described the factors influencing the production ratio of surfactin congeners. Peypoux and Michel (1992) reported that the addition of l-Val or l-Ile to the culture medium enhances the substitution of Leu7 of surfactin A with Val7 (surfactin B). Furthermore, Peypoux et al. (1994) reported that the cultivation of the same strain in a medium with l-Leu or l-Ile as a nitrogen source enhances the substitution of Val4 of surfactin A with Ala, Leu, or Ile and Leu7 of surfactin A. Production ratio of surfactin congeners is influenced by exogenous amino acids in the culture medium.

Among surfactin A, B and C, there are a few reports described about biological functions in the ecology of *Bacillus* spp. Aleti et al. (2016) reported that a surfactin A-producing *Bacillus* strain utilizes exogenous surfactin A for its pellicle formation and plant colonization, whereas significantly low by exogenous surfactin C. Moreover, Henry et al. (2011) reported that elicitor activity of surfactin A to induce disease resistance in tobacco cells is relatively higher compared with surfactin B or C. These findings strongly suggest that the fundamental insights into surfactin congener biosynthesis in *Bacillus* spp. is important to use these beneficial strains.

In this study, we evaluate the elution profiles of surfactin obtained via liquid chromatography-mass spectrometry (LC–MS) analysis. The elution profiles varied among the surfactin-producing isolates under the same culture conditions, suggesting that the production ratio of surfactin congeners is also influenced by exogenous factors. Therefore, we aimed to identify the endogenous factors affecting the production ratio of surfactin congeners.

## Materials and methods

### Strains and plasmids

All strains and plasmids used in this study are listed in Table 1.

**Table 1:** List of strains and plasmids used in this study

Strain/plasmid	Genotype/description
Strains	
*Escherichia coli*	
JM109	*mcrA recA1 supE44 endA1 hsdR17* (*rK*^–^*mK*^+^) *gyrA96 relA1 thi* ∆(*lac-proAB*) *F’* [*traD36 proAB + lacIq lacZ ∆M15*]
*Bacillus subtilis*	
168	*trpC2 sfp*^-^
JCM1465	Prototroph (type species for Bacillus subtilis)
168S	*trpC2 sfp*^+^
168S *srfAA7* < > *srfCA7*	*trpC2 sfp* ^+^ *srfAA7 < > srfCA7*
NB22	Undomesticated wild strain
*Bacillus* sp.	
TUA12	Undomesticated wild strain
TU18	Undomesticated wild strain
TUA24	Undomesticated wild strain
TCG15	Undomesticated wild strain
Ptrs2	Undomesticated wild strain
ATCC21556	Undomesticated wild strain
ATCC21770	Undomesticated wild strain
ATCC27505	Undomesticated wild strain
Plasmids	
pE194	EmR *ori*pE194 (temperature-sensitive)
pUC19	AmpR *lacZ* *ori*pUC19
pEUCT	pUC19 carrying *ori*pE194 EmR fargment (integrated by HindIII)
pEUCT::*sfp*	pEUCT carrying *sfp*JCM1465 (integrated by XbaI and KpnI)
pEUCT::*srfACA7*	pEUCT carrying *srfACA7*Ptrs2 (integrated by XbaI and KpnI)

## Media and culture conditions

No. 3S medium (Asaka and Shoda 1996) (10 g L^−1^ PolypeptonS [Nihon Pharmaceutical, Tokyo Japan], 10 g L^−1^ glucose, 1 g L^−1^ KH_2_PO_4_, and 0.5 g L^−1^ MgSO_4_‧7H_2_O) was used for the determination and purification of cLPs. Luria–Bertani medium (10 g L^−1^ tryptone, 0.5 g L^−1^ yeast extract, and 10 g L^−1^ NaCl) was used for genetic manipulation using *Escherichia coli*. For transformation of *B. subtilis*, we used SP I medium (SP I salt [2 g L^−1^ (NH_4_)_2_SO_4_, 14 g L^−1^ K_2_HPO_4_, 6 g L^−1^ KH_2_PO_4_, 1 g L^−1^ Na-Citrate ‧H_2_O, and 0.2 g L^−1^ MgSO_4_‧7H_2_O] with 0.1% of 50% glucose and 0.1% Casamino acids/yeast Extract [20 g L^−1^ Casamino acids and 100 g L^−1^ yeast extract]) and SP II medium (SP I medium with 0.1% of 50 mM CaCl_2_ and 0.1% of 250 mM MgCl_2_) developed by TaKaRa Bio (Shiga, Japan).

To select the transformants, 100 µg mL^−1^ ampicillin for *E. coli* and 20 µg mL^−1^ erythromycin for *B. subtilis* were added to the growth medium.

### Surfactin extraction and purification

Surfactin producing strains were cultivated with 30 mL of No. 3S medium at 30 °C for three days. The cultures were centrifuged at 8,000 × *g* for 10 min, and 20 mL of ethyl acetate was added to the supernatants twice to extract surfactins. The organic phase was collected and dried using rotary (N-1110; EYELA, Tokyo, Japan) and centrifugal evaporators (VC-36; TAITEC, Tokyo, Japan). The residue was dissolved in methanol and subjected to LC–MS and LC–MS/MS analyses.

Organic phase was subjected to silica gel and ODS column chromatography with FPLC (Purif-espo-Rp2; SHOKO SCIENTIFIC, Kanagawa, Japan) to purify the surfactins. For silica gel chromatography, isocratic and gradient elution were performed at a constant flow rate of 20 mL min^−1^ with solvent A as methanol and solvent B as ethyl acetate as follows: Time = 0–5 min, 0:100 (%A:%B); 20 min, 10:90; 25 min, 10:90; 50 min, 50:50; 55 min, 100:0; Column = Purif-pack easy SI 60 mm; Size: 60 (SHOKO SCIENTIFIC, Kanagawa, Japan). ODS chromatography was used for stepwise elution at a flow rate of 30 mL min^−1^ with solvent A as SPW and solvent B as methanol as follows: Time = 0–5 min, 20:80 (%A/%B); 5–15 min, 10:90; 15–25 min, 0:100; Column: Purif-pack ODS 100 mm; size: 120 (SHOKO SCIENTIFIC).

### Analysis of amino acid composition

To determine the amino acid composition of the surfactin analogs, the surfactins were hydrolyzed, and their amino acid ratios were analyzed via LC–MS. Briefly, 10 mg surfactin was added to 2 mL of 6 M HCl and heated at 110 °C for 24 h (Peterson et al. 1999). Hydrolyzed samples were washed four times with 50% MeOH and dissolved in 0.1 N HCl. LC–MS analysis was performed using the Infinity II 1260 HPLC system coupled to the single quadrupole mass spectrometer, Agilent Infinity Lab LC-MSD (Agilent Technologies, Santa Clara, CA, USA), equipped with atmospheric pressure ionization electrospray (API-ES) in the positive mode. The Intrada Amino Acid column (100 × 3 mm; Imtakt, Kyoto, Japan) was used at 40 °C to separate the amino acids. Solvent A was acetonitrile containing 0.1% (v/v) formic acid and Solvent B was acetonitrile and 100 mM ammonium formate (20:80) containing 0.3% (v/v) formic acid. The test was performed at a flow rate of 0.6 mL min^−1^ as follows: at 0–4 min, 20 (B%); at 4–14 min, 20–100; at 14–25 min, 100. To plot the calibration curve, we used a type H Amino Acid Mixture Standard Solution (FUJIFILM Wako Pure Chemical Corporation, Osaka, Japan).

### LC–MS analysis of surfactins

Quantification of cLPs was performed using the LC–MS system, Infinity II 1260 HPLC, coupled to a single quadrupole mass spectrometer (Agilent Infinity Lab LC-MSD; Agilent Technologies, Santa Clara, CA, USA) equipped with API-ES in the positive mode. Chromatographic separation was performed using the C18 column (UP 1.8 μm, 2.1 mm × 50 mm; Agilent Technologies, Santa Clara, CA, USA). The flow rate was maintained at 0.4 mL min^−1^ with solvent A as SPW containing 0.1% (v/v) formic acid and solvent B as acetonitrile containing 0.1% (v/v) formic acid (40:60 = %A:%B) for 50 min.

### LC-electrospray ionization (ESI)-MS/MS analysis

ESI–MS/MS coupled with collision-induced dissociation and helium collision gas was used for further identification of the amino acid sequences of cLPs. The selected precursor ions were acquired via auto-LC–ESI–MS/MS, and the data were analyzed using Accurate-Mass Q-TOF LC/MS with Agilent 6530 (Agilent Technologies, Santa Clara, CA, USA). Chromatographic separation was performed using Inert Sustain AQ-C18 (3 µm, 1.0 × 150 mm; GL Sciences, Tokyo, Japan). The gradient was maintained a constant flow rate of 0.07 mL min^−1^ with solvent A as ultrapure water (UPW) containing 0.1% (v/v) formic acid and solvent B as acetonitrile containing 0.1% (v/v) formic acid as follows: 0 min, 70:30 (%A:%B); 1 min, 70:30; 13 min, 0:100; 15 min, 0:100. These data were analyzed using the Agilent Mass Hunter Qualitative Analysis Software (version B. 06.00).

### Draft genome sequencing and analysis of surfactin biosynthesis genes

A genomic DNA library was prepared using the Illumina Nextera DNA Flex Library Prep Kit (Illumina, San Diego, CA, USA), according to the manufacturer’s instructions. Whole genome sequencing was performed using a paired-end sequencing strategy (2 × 300 bp) on an Illumina MiSeq sequencing platform (Illumina). Adapter sequences and low-quality regions were trimmed using Trim Galore! v.0.6.4, with default settings (https://www.bioinformatics.babraham.ac.uk/projects/trim_galore/). De novo assembly of trimmed genome sequences was performed using SPAdes v.3.11.1 (Bankevich et al. 2012). Auto-annotation of genome sequences was performed using the DDBJ Fast Annotation and Submission Tool (DFAST) (Tanizawa et al. 2016).

Genome sequencing data for strains ATCC21556 (DRR575793), ATCC21770 (DRR575794), ATCC27505 (DRR575795), NB22 (DRR575796), Ptrs2 (DRR575797), TCG15 (DRR575798), TUA12 (DRR657958), TUA18 (DRR575799), and TUA24 (DRR575800) have been deposited in the DDBJ database, respectively.

To identify the surfactin biosynthesis genes, a homology search was conducted via comparison with the *srfA* operon in *B. subtilis* 168 using in silico analysis (In Silico Molecular Cloning, In Silico Biology, Japan). Multiple sequence alignment was performed using CLUSTALW, a Maximum Likelihood tree was constructed using MEGA 11 (RRID:SCR_023017) and edited by iTOL (Letunic and Bork 2024).

### Average nucleotide identity (ANI) analysis

To identify the *Bacillus* species used in this study, we performed ANI analysis (Konstantinidis and Tiedje 2005; Goris et al. 2007) using DDBJ DFAST.

### *B. subtilis* strain construction

*Bacillus subtilis* 168S (*sfp* +) was generated by substituting the *sfp* gene in *B. subtilis* 168 (which lacked functionality due to a frameshift mutation (Nakano et al. 1992)) with the gene from *B. subtilis* JCM 1465.

Temperature-sensitive replication vector pEUCT was used for substitution. To construct pEUCT, the gene cassette, comprising an origin capable of replication and conferring erythromycin resistance to *Bacillus* strains, was amplified using pE194 (Iordănescu 1976) via polymerase chain reaction (PCR) using the primers, pE194-Emr-rep-F and pE194-Emr-rep-R. The PCR products were digested with HindIII and ligated into pUC19 (Norrander et al. 1983). The resulting ligation products were transformed into *E. coli* JM109. Subsequently, to construct the *sfp* substitution vector pEUCT::*sfp*, the *sfp* gene from *B. subtilis* JCM1465 chromosomal DNA was amplified via PCR using primers 168sfp_del1_f_XbaI and 168sfp_del3_r_KpnI, digested with XbaI and KpnI, and ligated into pEUCT. The resulting ligation products were transformed into *E. coli* JM109 (Yanisch-Perron et al. 1985).

*B. subtilis* 168S (*sfp* +) was generated using a two-step replacement method. Briefly, the pEUCT::*sfp* substitution vector was transformed into *B. subtilis* 168 using the protocol developed by TaKaRa Bio. *B. subtilis* 168 carrying pEUCT::*sfp* was cultivated at 42 °C to induce single crossover. The temperature-sensitive origin of pEUCT halted replication at 42 °C, facilitating the insertion of homologous sequences into the genome through a single crossover. In the second step, transformants were cultivated at 37 °C for 24 h without erythromycin. Colonies with *sfp*-substituted mutants exhibited an erythromycin-sensitive phenotype that was used to identify the target genotype. Sensitive clones were verified via PCR.

To generate 168S A7 domain-substituted mutants, an A7 domain substitution vector, pEUCT::*srfC A7*, was constructed. Briefly, 0.8-kbp fragments of the upstream and downstream regions of the 168S srfA7 domain were amplified. Then, DNA fragments of the *srfC* A7 domain region of Ptrs2 were amplified. These fragments were used as templates for the overlapping PCR products. The resulting PCR products were digested with XbaI and KpnI and ligated into pEUCT. Finally, 168S cells were transformed as described above.

## Results

### Elution profiles of surfactins determined via LC–MS analysis

**Fig. 3:**
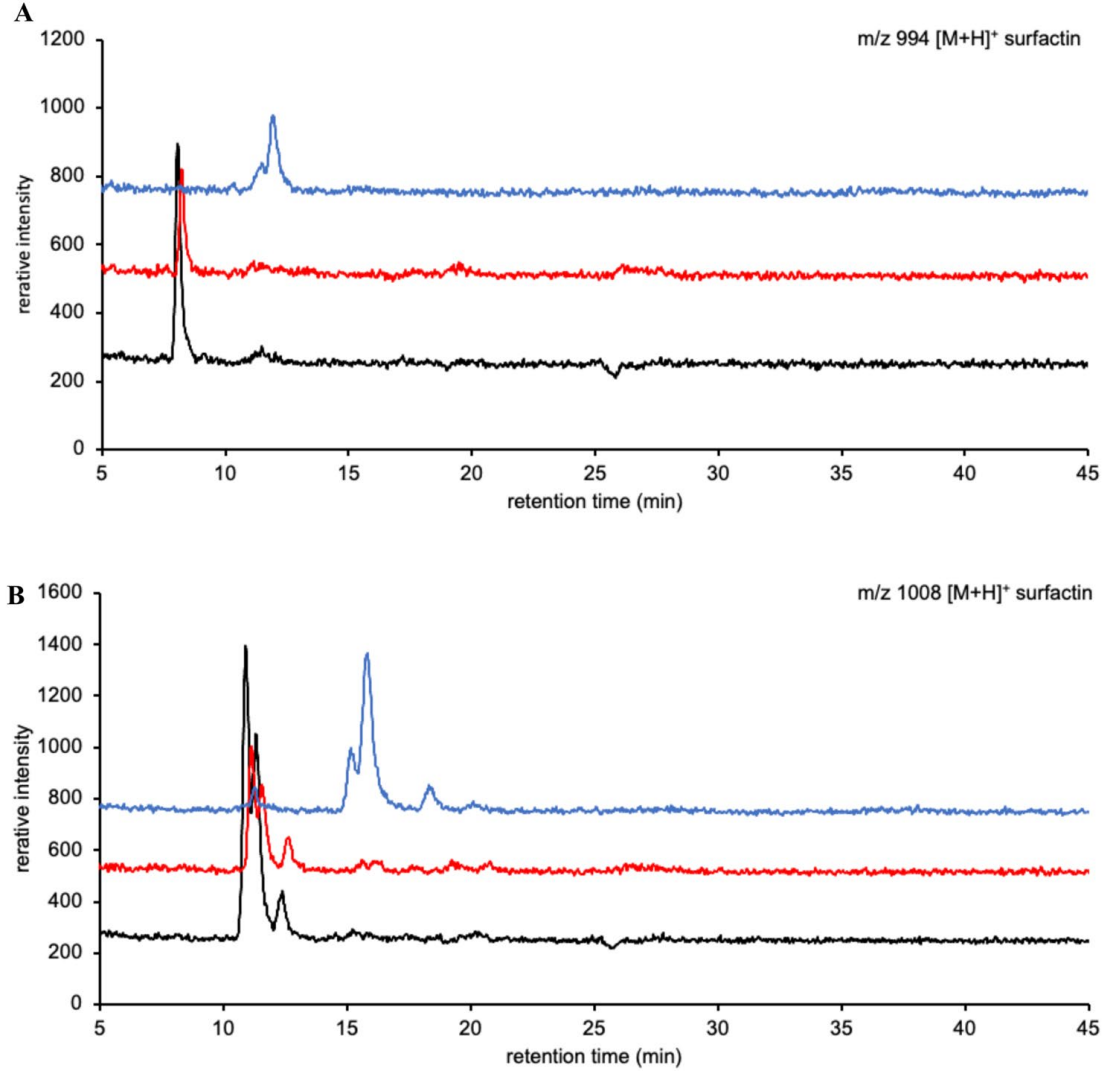

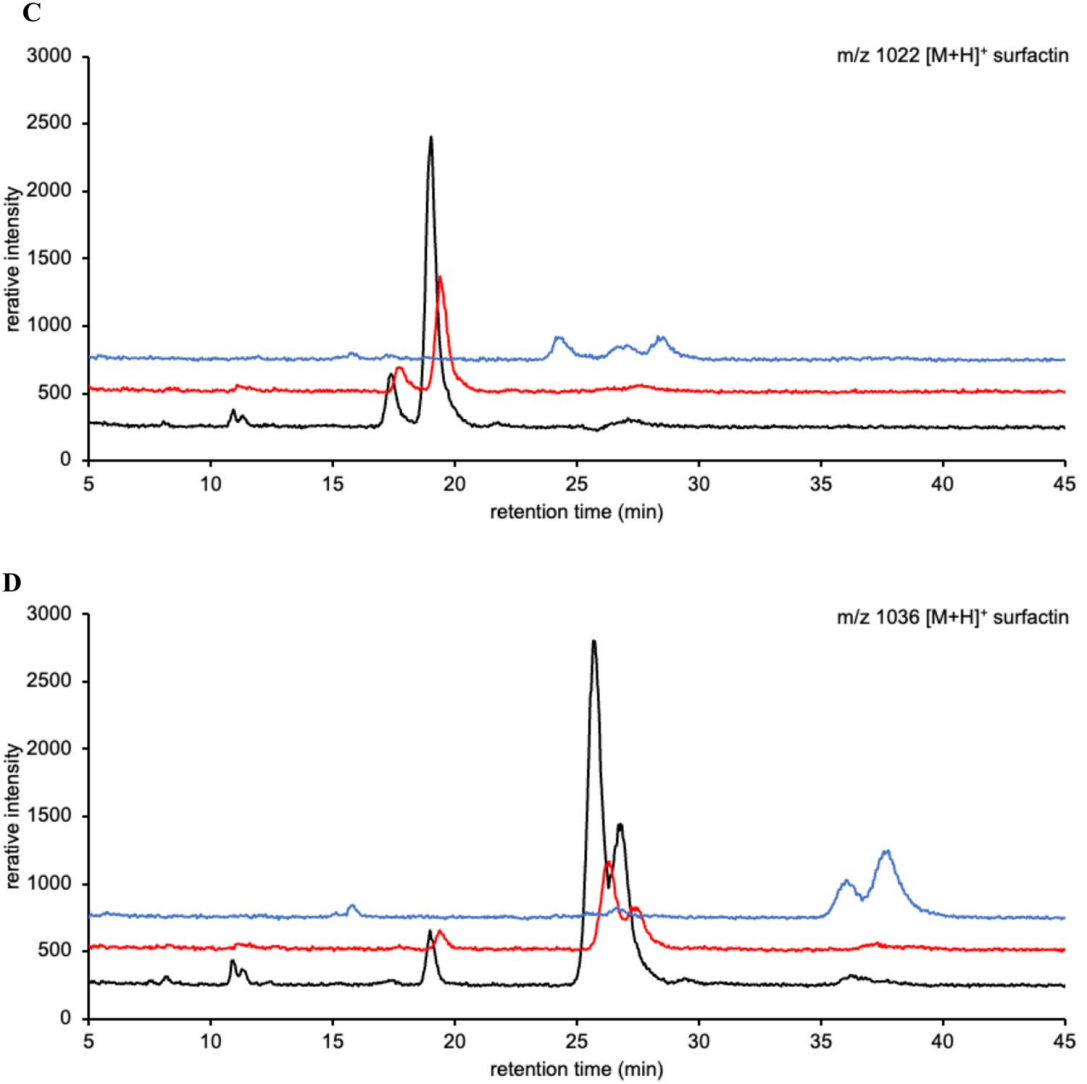
Elution profiles of surfactins. (**A**, **B**, **C**, and **D**) Mass chromatograms at *m/z* [M + H].^+^ 994, 1008, 1022, and 1036, respectively. Black line: JCM 1465; Red line: TUA12; Blue line: Ptrs2

Intense surfactin signals were observed as protonated ions via LC–MS. The ions detected at *m*/*z* 994, 1008, 1022, and 1036 corresponded to [surfactin A/C + H]^+^ containing C_12_, C_13_, C_14_, and C_15_ fatty acid chains or [surfactin B + H]^+^ containing C_13_, C_14_, C_15_, and C_16_ fatty acid chains, respectively. Based on the LC–MS elution profiles, the 10 surfactin-producing strains could be divided into two types (Fig. 3 and Supplementary Fig. S1). The difference in elution profiles was possibly due to the variety in surfactin congeners.

### Structural identification of surfactins

**Fig. 4:**
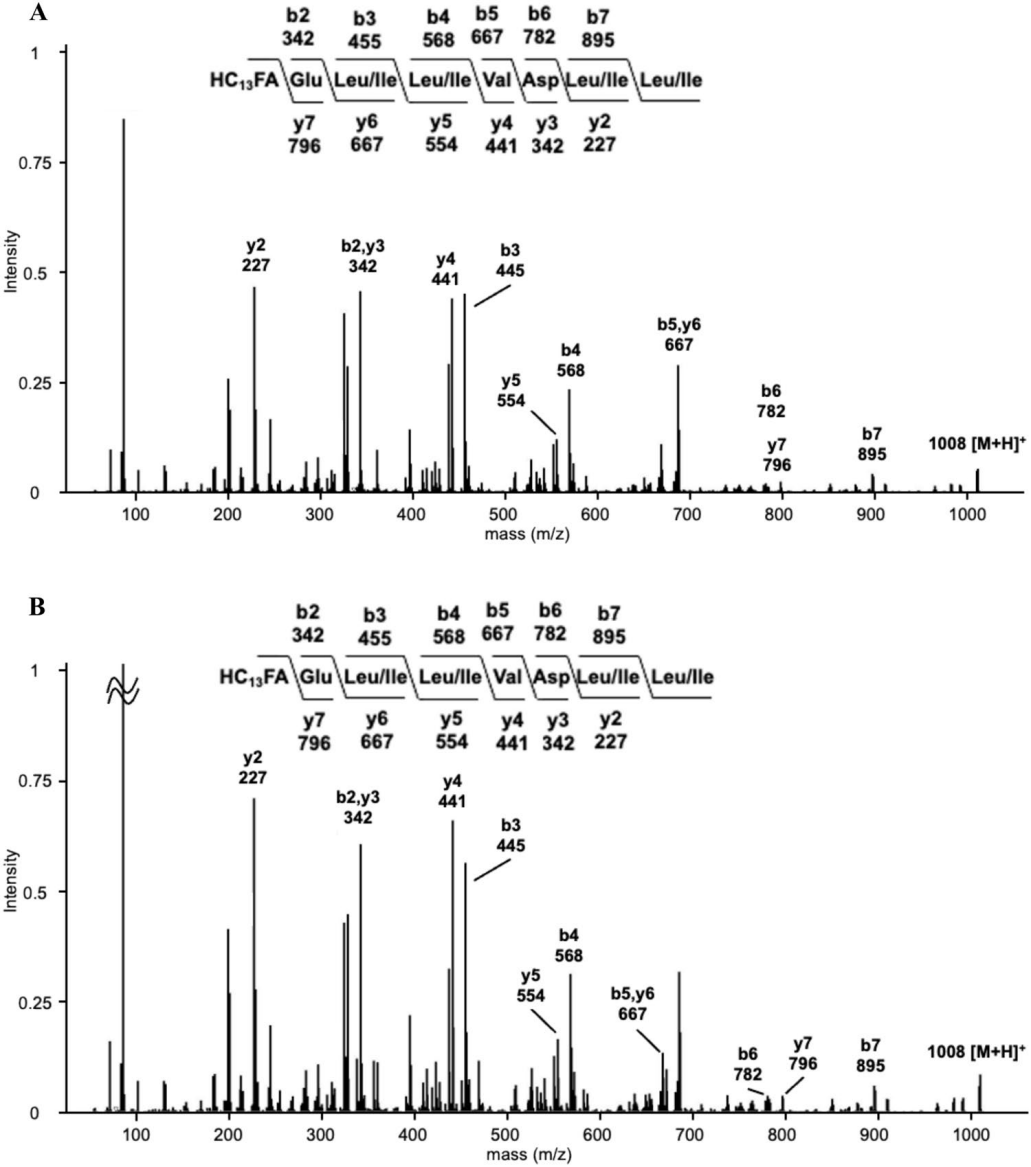
Liquid chromatography-tandem mass spectrometry (LC–MS/MS) analysis of surfactins. (**A**) Chromatogram at *m/z* 1008 [M + H]^+^ for the surfactin isolated from TUA12. (**B**) Chromatogram at *m/z* 1008 [M + H].^+^ for the surfactin isolated from Ptrs2

To identify the primary structures of the peptide moieties in the two types of surfactins, we obtained MS^2^ spectra via LC–ESI–MS/MS analysis of purified surfactins. The MS^2^ spectra of the two types of surfactins were identical for each parent ion (Fig. 4A and B). The primary structure of the surfactin peptide moiety was Glu-Leu/Ile-Leu/Ile-Val-Asp-Leu/Ile-Leu/Ile, suggesting that the two types of surfactins are structural isomers. Moreover, some surfactin peaks corresponded to surfactin B for all isolates; however, these peaks accounted for approximately 0.5–9.6% of total surfactin (Supplementary Table 5).

To identify the amino acid composition of the peptide moiety of surfactin, purified surfactins were acid hydrolyzed, and the resulting free amino acids were quantified via LC–MS. The amino acid composition was Leu:Val:Glu:Asp = 3.9:0.9:1.0:1.1 for the surfactin derived from TUA12 and Leu:Ile:Val:Glu:Asp = 3.0:1.1:0.9:0.8:0.8 for the surfactin derived from Ptrs2 (Table 2).

**Table 2:** Amino acid compositions of surfactins

	TUA12	Ptrs2
Leu	4	3
Ile	0.2	1
Val	0.9	0.9
Glu	0.7	0.8
Asp	1.3	0.8

Our data revealed that the surfactin derived from TUA12 was surfactin A containing Glu-Leu-Leu-Val-Asp-Leu-Leu and that derived from Ptrs2 was formed by substituting Leu2, Leu3, Leu6, and Leu7 in surfactin A with Ile.

### Identification and phylogenetic analysis of the adenylation domain in *srf* operon

**Fig. 5:**
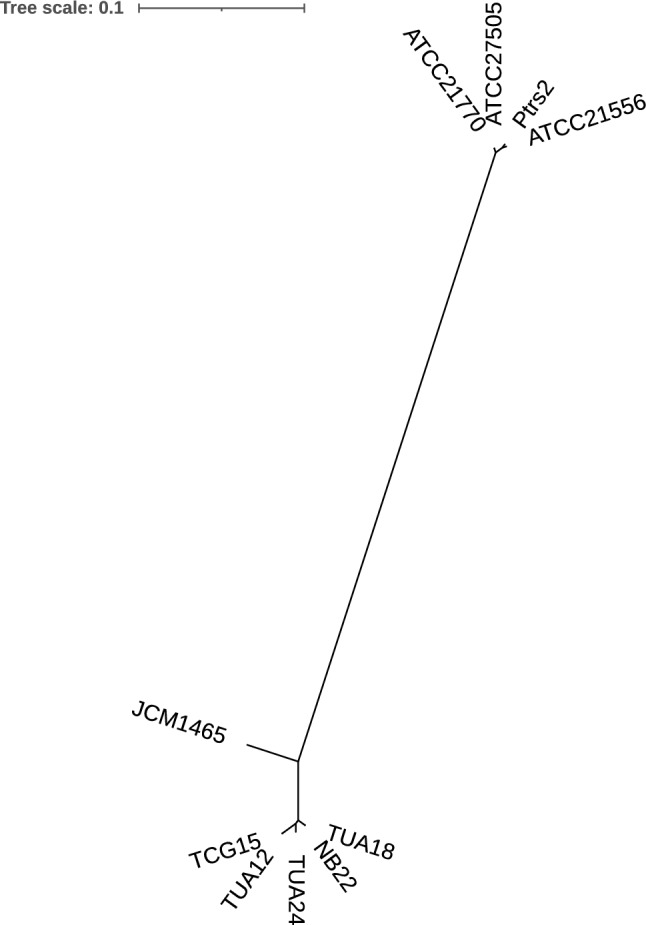
Phylogenetic tree of the A7 domain of *srfA* operon

Characteristics of the sequenced genomes are listed in Table 3. Adenylation domain in NRPS is responsible for the selection and recruitment of amino acids for peptide synthesis. Therefore, we compared the deduced amino acid sequences of each 7-adenylation domain predicted using antiSMASH (Blin et al. 2023) (RRID:SCR_022060) in the NRPS operon for surfactin biosynthesis (Supplementary Fig. S2). The adenylation domains 1–6 in *srfA* operon, which were responsible for selection of Glu1, Leu2, Leu3, Val4, Asp5, and Leu6 of surfactin A, respectively, showed more than 91% homology for each domain among the 10 tested strains, except for JCM1465 (Supplementary Table 1). The adenylation domain 7 for Leu7 of surfactin A showed lower homology (66.1–67.3%) between surfactin A-producing and other strains (Fig. 5), suggesting that the substitution of Leu with Ile occurred at Leu7 of surfactin A.

**Table 3:** Genomic features of the surfactin-producing *Bacillus* spp. analyzed in this study

Strain	Total length (bp)	GC content	Total number of contigs (bp)	Number of CDSs	Longest contig (bp)	Coding ratio (%)	N50 (bp)	Number of rRNAs	Number of tRNAs	Genome Coverage
ATCC21556	3,916,204	45.78	12	4004	2,054,301	88.7	2,054,301	6	78	314x
ATCC21770	3,950,795	46.01	24	4047	983,478	88.3	840,655	7	80	237x
ATCC27505	3,911,672	46.06	19	4002	980,673	88.4	604,035	5	79	247x
NB22	3,852,662	46.56	23	3673	1,087,749	88.1	876,367	7	80	280x
Ptrs2	3,831,383	46.05	18	3823	1,076,349	88.4	983,704	3	79	253x
TCG15	3,986,550	46.57	30	3873	1,614,700	89	258,825	5	82	222x
TUA12	3,999,878	46.36	25	3894	733,656	89.2	667,735	8	80	181x
TUA18	3,854,561	46.57	15	3677	2,003,870	89	2,003,870	7	80	217x
TUA24	4,052,407	46.11	25	3980	844,712	89.1	430,979	7	81	325x

### Evaluation of the adenylation domain for surfactin congener biosynthesis

To evaluate the 1.2-kbp adenylation domain 7 for the two types of surfactin biosynthesis, we obtained adenylation domain 7 substituted mutants of the surfactin-producing *Bacillus* strains. Strain 168 is a laboratory strain derived from JCM1465, a surfactin A producer. Strain 168 lacks surfactin biosynthesis due to the frame shift of *sfp*, which is involved in surfactin biosynthesis. Prior to the adenylation domain 7 substitution, we obtained *sfp* substitution mutant 168S and confirmed surfactin A production. Then, we obtained mutants of adenylation domain 7 substituted with 168S and 168S, *srfA A7* < > *srfAC A7*.

**Fig. 6:**
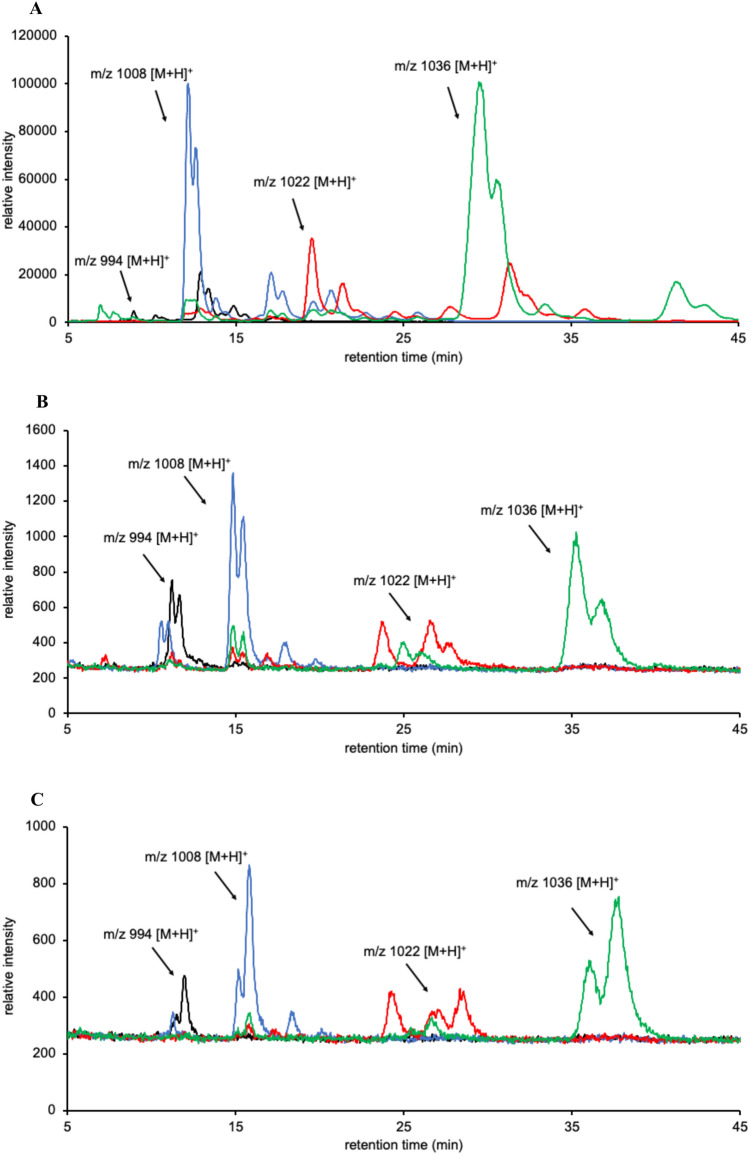
LC–MS chromatograms of surfactins isolated from the 168 mutant and ptrs2 strains of *Bacillus subtilis*. (**A**) Surfactin A isolated from 168S. (**B**) Surfactin C isolated from 168S srfAA7 < > srfCA7. (**C**) Surfactin C isolated from Ptrs2. Black line: *m/z* [M + H]^+^ 994 surfactin; Blue line: *m/z* [M + H]^+^ 1008 surfactin; Red line: m/z [M + H]^+^ 1022 surfactin; Green line: *m/z* [M + H]^+^ 1036 surfactin

Elution profile of surfactin produced by 168S *srfA A7* < > *srfAC A7* was identical to that of Ptrs2 (Fig. 6), and the amino acid composition of surfactin derived from 168S *srfA A7* < > *srfAC A7* was Leu:Ile:Val:Glu:Asp = 3.0:1.0:0.9:0.7:1.1, which corresponded to the elution profile of surfactin C. Therefore, surfactin conger produced by Ptrs2, ATCC 21556, ATCC 21770, and ATCC 27505 was determined to be surfactin C, which was formed by substituting Leu7 in surfactin A with Ile.

### Phylogenetic analysis of NRPS genes for surfactin biosynthesis

*srfA* operon encoding NRPS genes for surfactin biosynthesis consisted of four ORFs, *srfAABCD*, in *B. subtilis* JH642 (Galli et al. 1994). We conducted phylogenetic analyses of the deduced amino acid sequences of each ORF of the 10 tested strains.

**Fig. 7:**
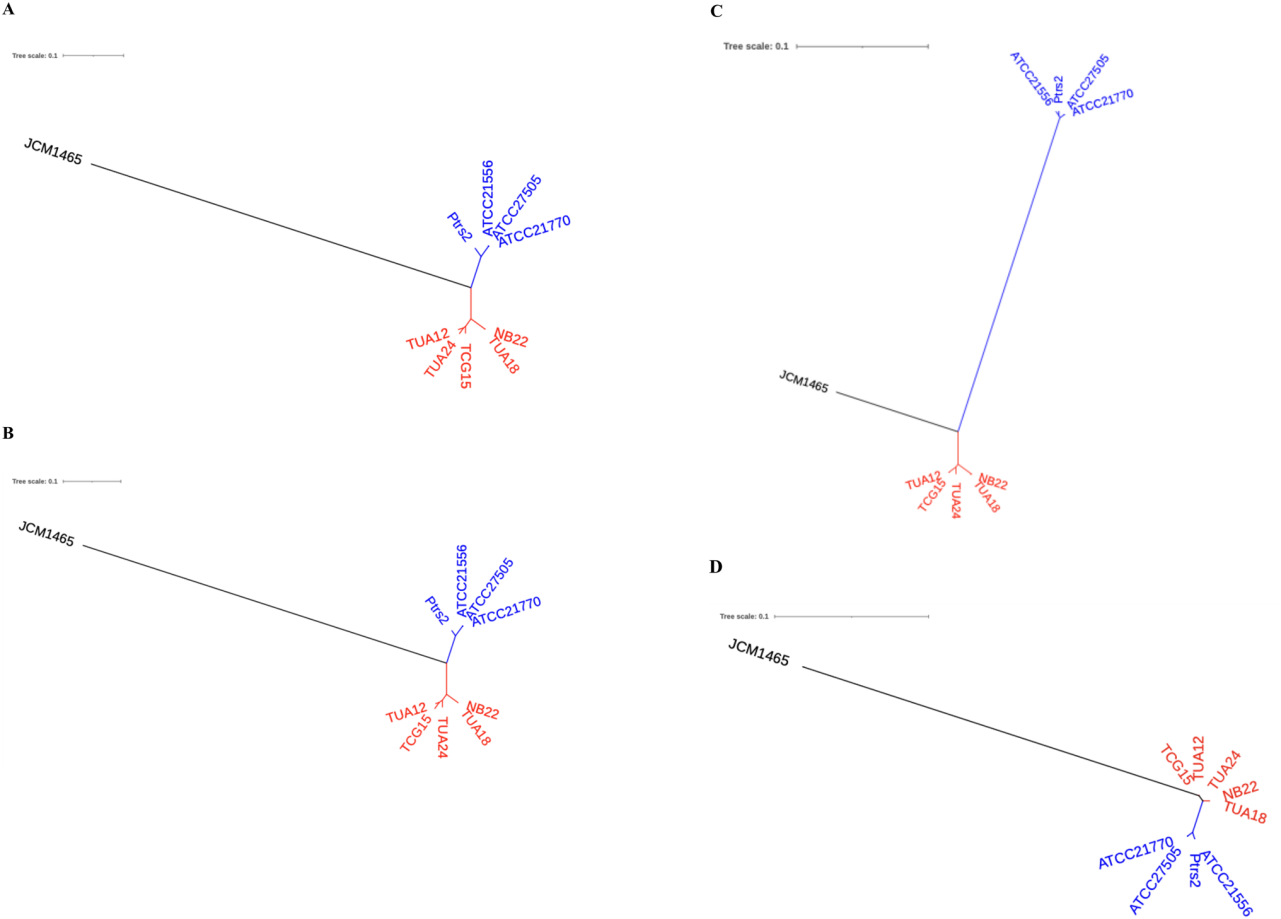
Phylogenetic analysis of the open reading frames (ORFs) in *srfA* operon. (**A**) *srfAA*. (**B**) *srfAB*. (**C**) *srfAC*. (**D**) *srfAD*

For each ORF, the 10 strains were divided into two phylogenetic groups and JCM 1465. Each ORF showed high homology in surfactin A-producing strains (97.4–100%), except for JCM 1465, and surfactin C-producing strains (> 98.2%). *srfAA, B* and D of JCM 1465 showed low homology (73.4–74.4%) with the other nine strains (Fig. 7A, B and D; Supplementary Table 2). *srfAC*, which encodes adenylation domain 7, showed relatively high homology with surfactin A-producing strains (86.2–86.7%) and slightly less homology with with surfactin C producing strains (71.7–71.9%; Fig. 7C; Fig. S4).

These results clearly indicate that the NRPS operons encoding surfactin C biosynthesis conserved in ATCC 21556, ATCC21770, ATCC27505, and Ptrs2 are distinct from the *srfA* operon of JCM 1465 and the operon encoding surfactin A biosynthesis genes conserved in NB22, TUA12, TUA18, TUA24, and TCG15.

### Distribution of *srf* operon in *Bacillus* spp.

ANI is used to identify prokaryotes based on their genomic sequences. Here, we identified the test strains and evaluated their relationships with the *srf* operons (Supplementary Table 3).

No tested strains showed more than 95% ANI values with JCM 1465^T^. All the four surfactin A-producing strains (NB22, TUA12, TUA18, TUA24, and TCG15) were identified as *B*. *velezensis* with more than 95% ANI values with *B*. *velezensis* NRRL B-41580^T^. All the five surfactin C-producing strains (ATCC 21556, ATCC21770, ATCC27505, and Ptrs2) were identified as *B*. *amyloliquefaciens* with more than 95% ANI values with *B*. *amyloliquefaciens* DSM7^T^.

## Discussion

Surfactin is a secondary metabolite and a cLP. Many congeners, varying in the acyl chain length and structural isomers of the b-hydroxy fatty acid moiety and primary structure of the heptapeptide moiety, have been identified to date (Fig. 2).

In this study, we found that the 10 tested strains could be divided into two groups based on the elution profiles of surfactins. Chemical and mutagenesis analyses revealed that the differences in elution profiles were due to surfactins A and C.

Production ratio of surfactin congeners based on the primary structure of the heptapeptide moiety is influenced by exogenous amino acids. Peypoux and Michel (1992) reported that the production ratio of surfactin A:B was enhanced from 86:14 to 57:43 or 49:51 by adding 5 g L^−1^ l-Val or l-Ile to the basal medium, respectively. Bonmatin et al. (1995) observed surfactin B production after the addition of l-Val or l-Ile to the basal medium. In this study, we observed surfactin B production of approximately 3.4% in all tested strains (Supplementary Table 5). However, the production ratio of surfactin A and C in this study was different from that for surfactin B, and no other congeners were detected between surfactins A and C under the same culture conditions.

*srfA* operon, the NRPS operon for surfactin A biosynthesis consisting of *srfAABCD*, was characterized in this study. The deduced amino acid sequences of the four ORFs encoding the NRPS operon in JCM1465 showed low homology with those of the other nine strains. As JCM1465 contains the *srfA* operon, the other nine strains may contain the *srfAA* homologs with 93–100% homology for biosynthesis of surfactin A or C. *srfAC* homologs encoding an adenylation domain for 7th amino acid of heptapeptide were clearly divided into two clades of surfactin A- and surfactin C-producing strains with 72–100% homology. Therefore, the 10 tested strains can be divided into three groups of *srfA* operon homologs.

**Fig. 8:**
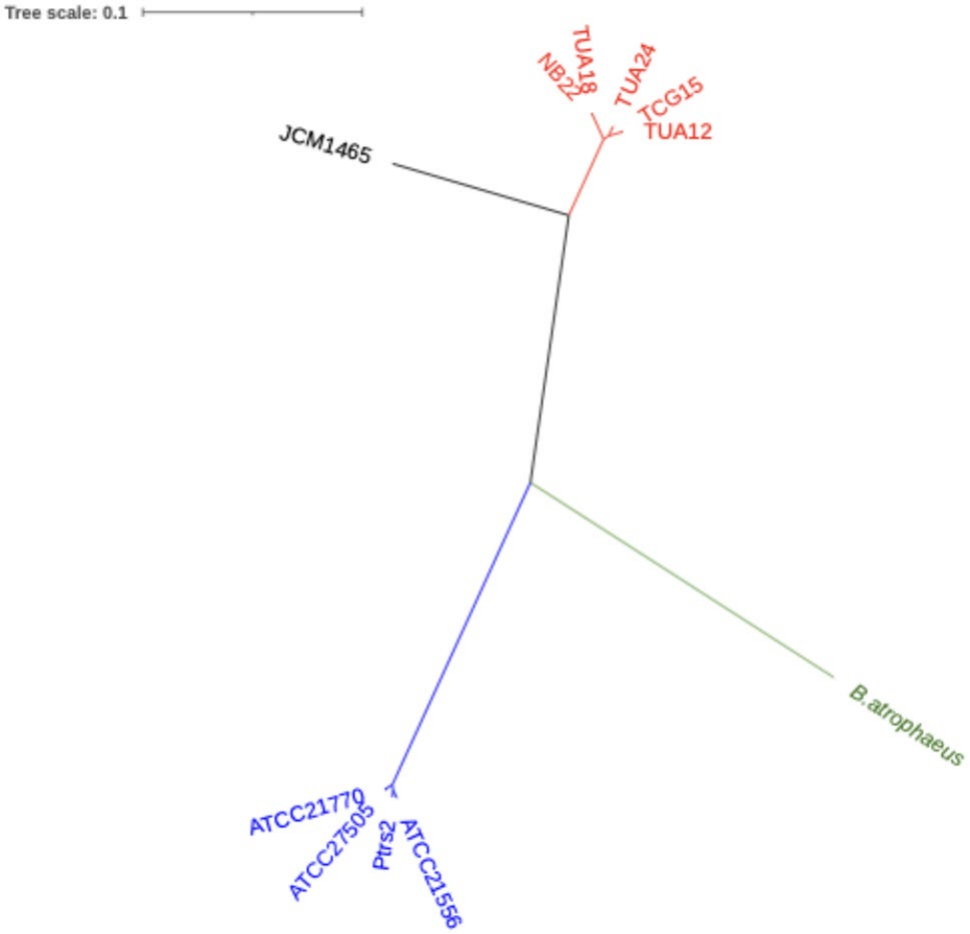
Phylogenetic tree of* srfC*

All tested strains, except *B. subtilis* JCM 1465^T^, were identified as *B. velezensis* or *B. amyloliquefaciens* via ANI analysis with more than 95% ANI values, and surfactin congener production was strictly limited *Bacillus* species. Aleti et al. ( 2016) reported that the NRPS operon for surfactin C is the *srfC* operon in *B. atrophaeus*. The NRPS operon for surfactin C biosynthesis in *B. amyloliquefaciens* is distinct from the *srfC* operon in *B. atrophaeus* (Fig. 8). To provide a clear and systematic nomenclature, we propose naming the NRPS operon in *B. velezensis*, which is responsible for surfactin A biosynthesis, as the *BvelsrfA* operon. Similarly, we propose naming the NRPS operons responsible for surfactin C biosynthesis in *B. amyloliquefaciens* and *B. atrophaeus* as the *BamysrfC* and *BatrsrfC* operons, respectively.

This nomenclature is based on the bacterial species in which these operons are conserved (Bvel for *B. velezensis*, Bamy for *B. amyloliquefaciens*, and Batr for B*. atrophaeus*) and the type of surfactin they are associated with (*srfA* for surfactin A and *srfC* for surfactin C). This classification aims to differentiate these operons from previously characterized surfactin biosynthetic pathways and improve clarity in future comparative studies.

BLASTP (Altschul et al. 1997) analysis revealed that ORF-C, a key determinant in differentiating surfactin A and C, forms species-specific clades in *B. amyloliquefaciens* and *B. velezensis* (Supplementary Fig. S3). This suggests that despite being closely related species, these bacteria have evolved distinct surfactin production profiles. Such molecular divergence may play a critical role in interspecies communication and niche specialization among *Bacillus* species.

Lozano-Andrade et al. (2025) proposed that surfactin reshapes the chemical landscape of microbial communities, thereby promoting niche differentiation. By modifying local chemical environments, surfactin-producing strains may establish unique ecological niches, reducing interspecies competition while maintaining stable coexistence.

These findings collectively highlight that surfactin is not merely a biosurfactant but also a key signaling molecule that influences microbial community structure and environmental adaptation. The differential production of surfactin congeners among *Bacillus* species likely reflects an evolutionary strategy for optimizing interactions within microbial ecosystems.

To facilitate the development of optimized microbial pesticides and novel biosurfactant-based products for industrial applications, further investigation into the antimicrobial activity and plant disease suppression effects of distinct cLP molecular species is highly anticipated. A deeper understanding of their structure–activity relationships will provide valuable insights into their functional diversity and potential utilization. Additionally, comparative genomic analyses focusing on horizontal gene transfer (HGT) events in surfactin biosynthesis genes could provide deeper insights into how these operons have evolved and diversified across *Bacillus* species.

## Supplementary Information

Below is the link to the electronic supplementary material.

Supplementary file1 (PDF 503 KB)

Supplementary file2 (PDF 89 KB)

Supplementary file3 (PDF 342 KB)

Supplementary file4 (XLSX 37 KB)

## Data Availability

Data is provided within the manuscript or supplementary information files.
