# Supplementary material for: Characterization of the NRPS operon homolog for surfactin A and surfactin C synthesis in Bacillus spp
Source: Arch Microbiol. 2025 May 29;207(7):161. doi: 10.1007/s00203-025-04341-z (PMC12122625; doi:10.1007/s00203-025-04341-z)
Supplement: Supplementary file 1 — Supplementary file1 (PDF 503 KB) [file 203_2025_4341_MOESM1_ESM.pdf]

Characterization of the NRPS operon homolog for surfactin synthesis in *Bacillus* spp., Archives of Microbology,

Kojiro Ito *et al*; Corresponding author: Kenji Yokota, Tokyo University of Agriculture, yokota@nodai.ac.jp

Supplementary information

A

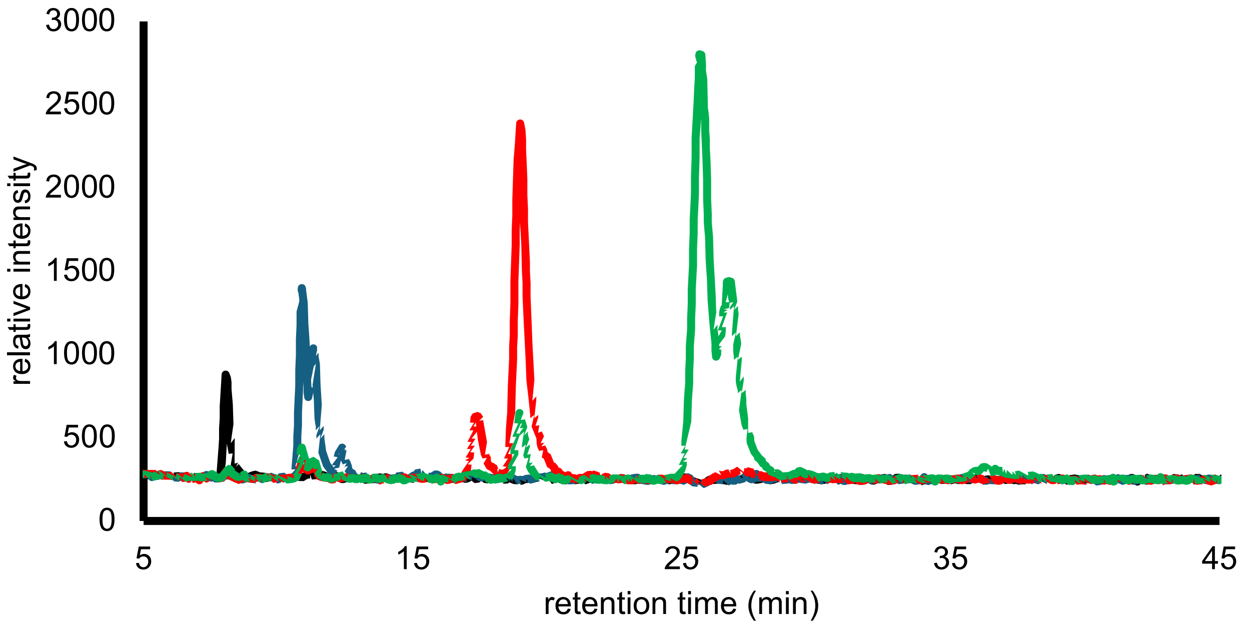

B

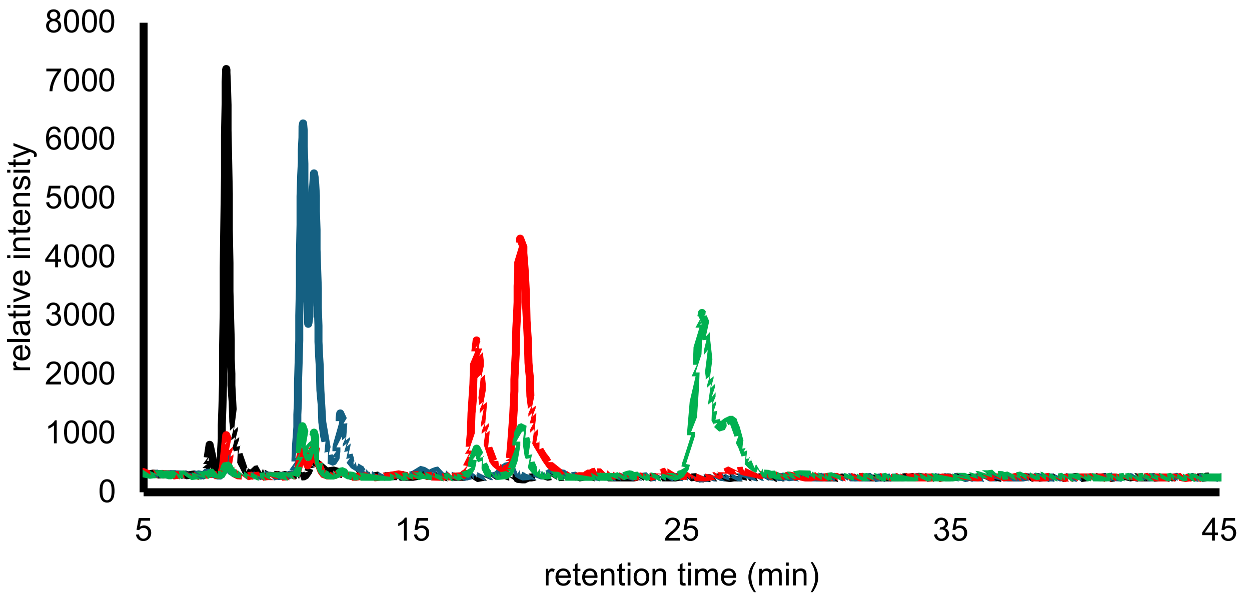

C

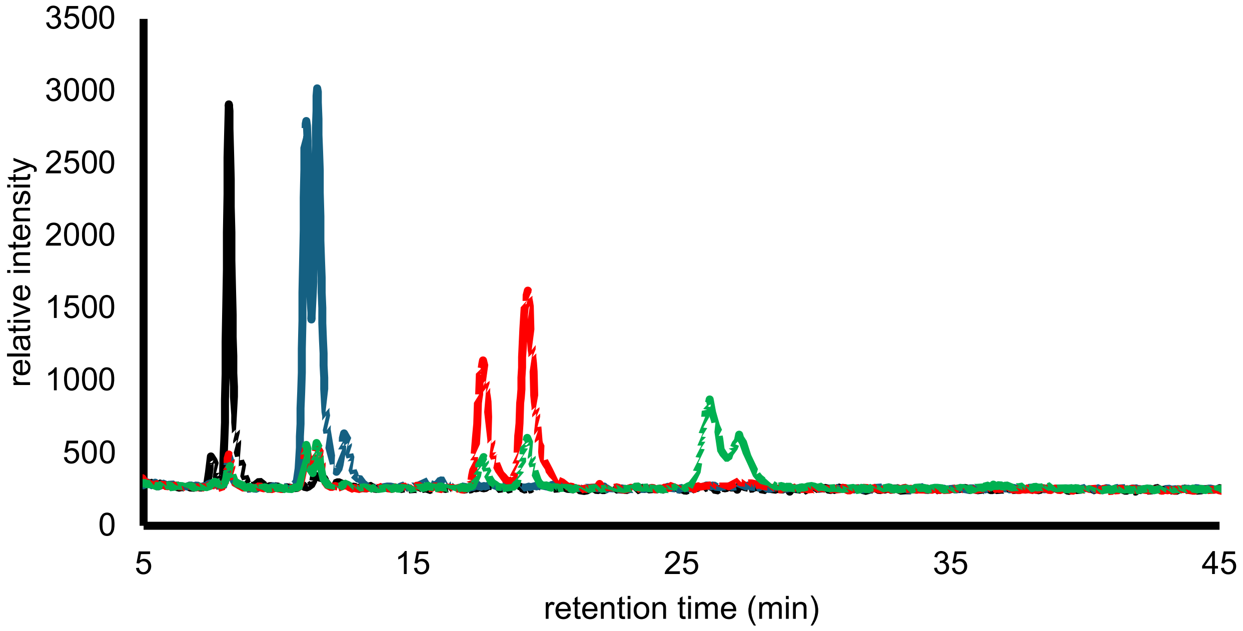
3500
3000
2500
2000
1500
1000
500
0
relative intensity
5
15
25
35
45
retention time (min)

D

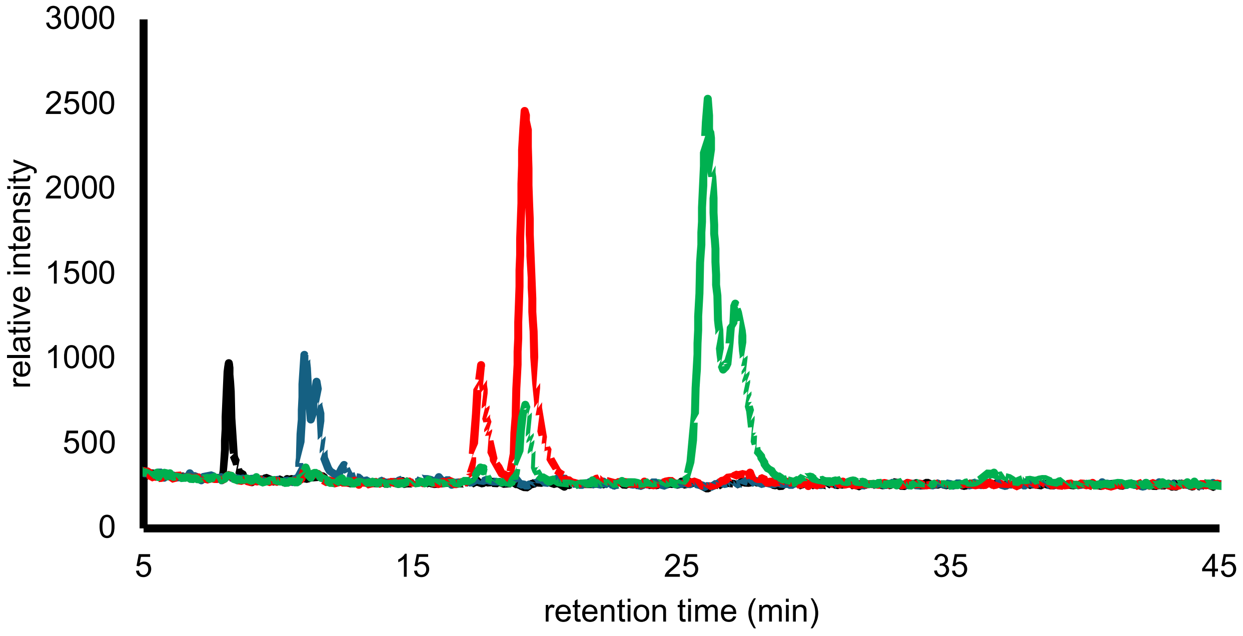
3000
2500
2000
1500
1000
500
0
relative intensity
5
15
25
35
45
retention time (min)

E

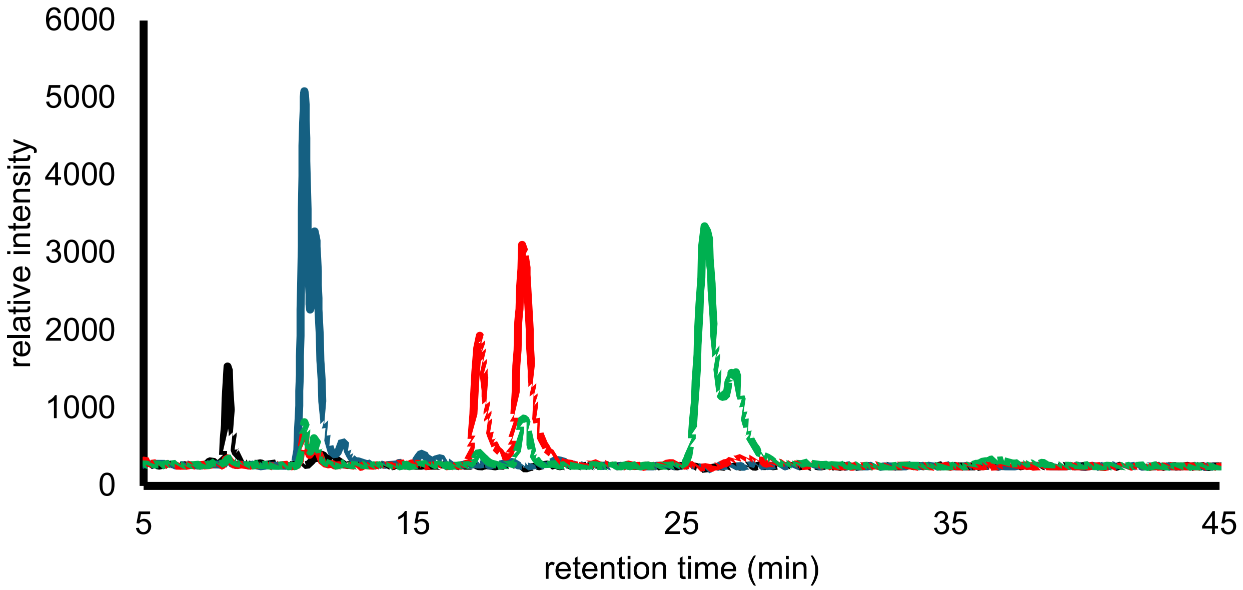
6000
5000
4000
3000
2000
1000
0
relative intensity
5
15
25
35
45
retention time (min)

F

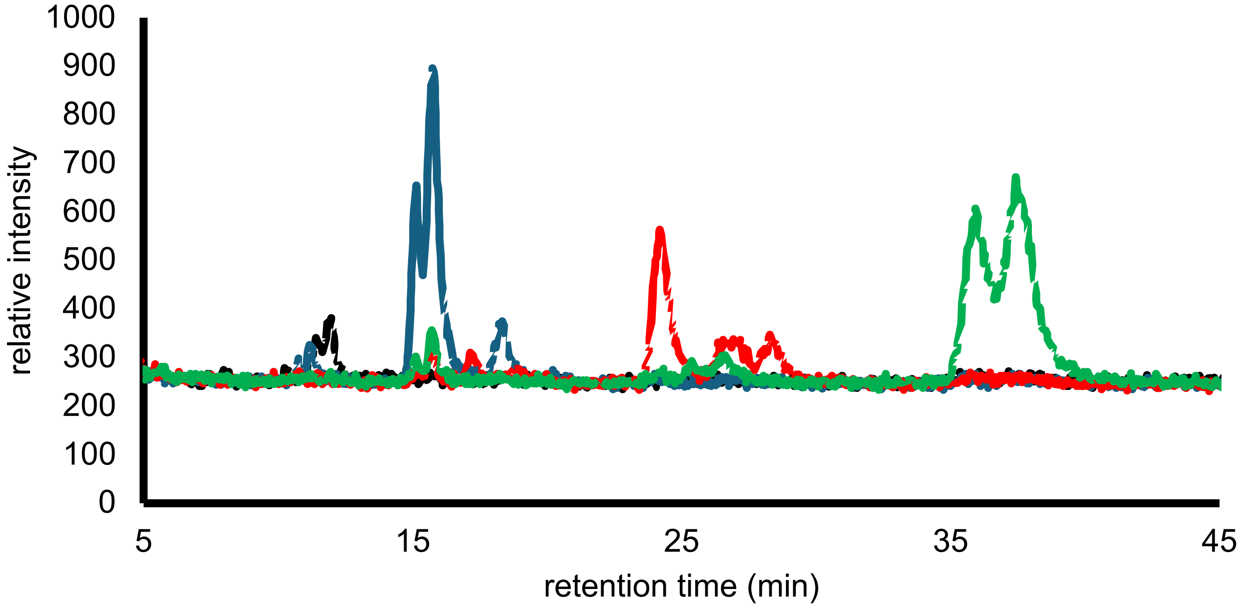
1000
900
800
700
600
500
400
300
200
100
0
relative intensity
5
15
25
35
45
retention time (min)

G

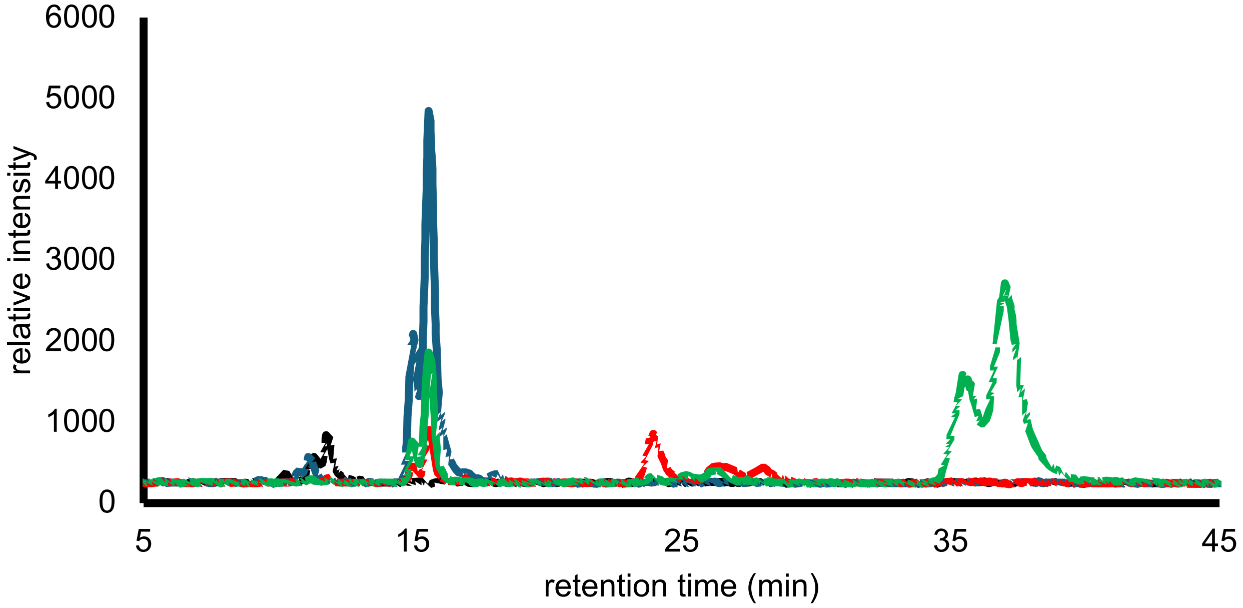

H

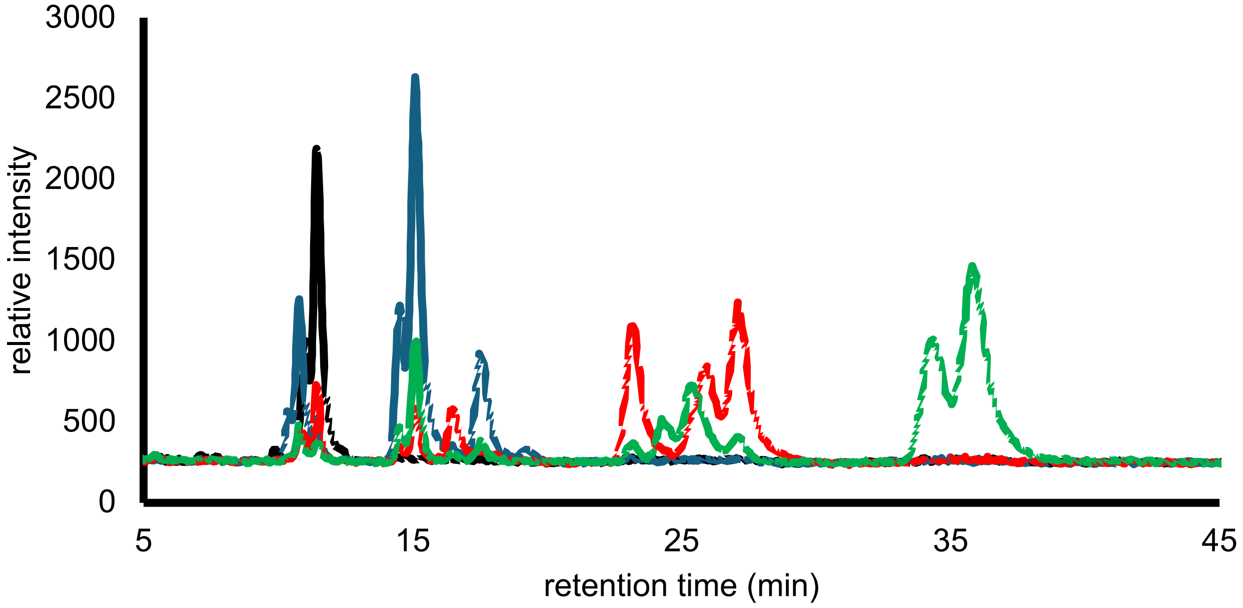

SI. 1: LC-MS chromatograms of surfactin derived from Bacillus strains

A: *B. subtilis* JCM1465

B: *B.velezensis* NB22

C: *B.velezensis* TUA18

D: *B.velezensis* TUA24

E: *B.velezensis* TCG15

Kojiro Ito *et al*; Corresponding author: Kenji Yokota, Tokyo University of Agriculture, yokota@nodai.ac.jp

F: *B. amyloliquefaciens* ATCC 21556
G: *B. amyloliquefaciens* ATCC 21770
H: *B. amyloliquefaciens* ATCC 27505
