# Supplementary material for: Characterization of the NRPS operon homolog for surfactin A and surfactin C synthesis in Bacillus spp
Source: Arch Microbiol. 2025 May 29;207(7):161. doi: 10.1007/s00203-025-04341-z (PMC12122625; doi:10.1007/s00203-025-04341-z)
Supplement: Supplementary file 2 — Supplementary file2 (PDF 89 KB) [file 203_2025_4341_MOESM2_ESM.pdf]

Characterization of the NRPS operon homolog for surfactin synthesis in *Bacillus* spp., Archives of Microbiology,

Kojiro Ito *et al*; Corresponding author: Kenji Yokota, Tokyo University of Agriculture, yokota@nodai.ac.jp

Supplementary information

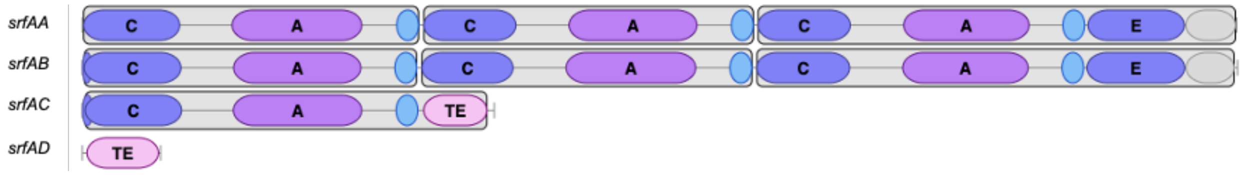

Supplementary Fig. S2: antiSMASH analysis to predict adenylation domain into *srfA* operon
C: Condensation domain
A: Adenylation domain
TE: Thioesterase domain
Blue circle: Peptide carrier protein = Thiolationdomain
Gray circle: Epimerization domain
