# Supplementary material for: Characterization of the NRPS operon homolog for surfactin A and surfactin C synthesis in Bacillus spp
Source: Arch Microbiol. 2025 May 29;207(7):161. doi: 10.1007/s00203-025-04341-z (PMC12122625; doi:10.1007/s00203-025-04341-z)
Supplement: Supplementary file 3 — Supplementary file3 (PDF 342 KB) [file 203_2025_4341_MOESM3_ESM.pdf]

Characterization of the NRPS operon homolog for surfactin synthesis in *Bacillus* spp., Archives of Microbiology,

Kojiro Ito *et al*; Corresponding author: Kenji Yokota, Tokyo University of Agriculture, yokota@nodai.ac.jp

Supplementary information

A

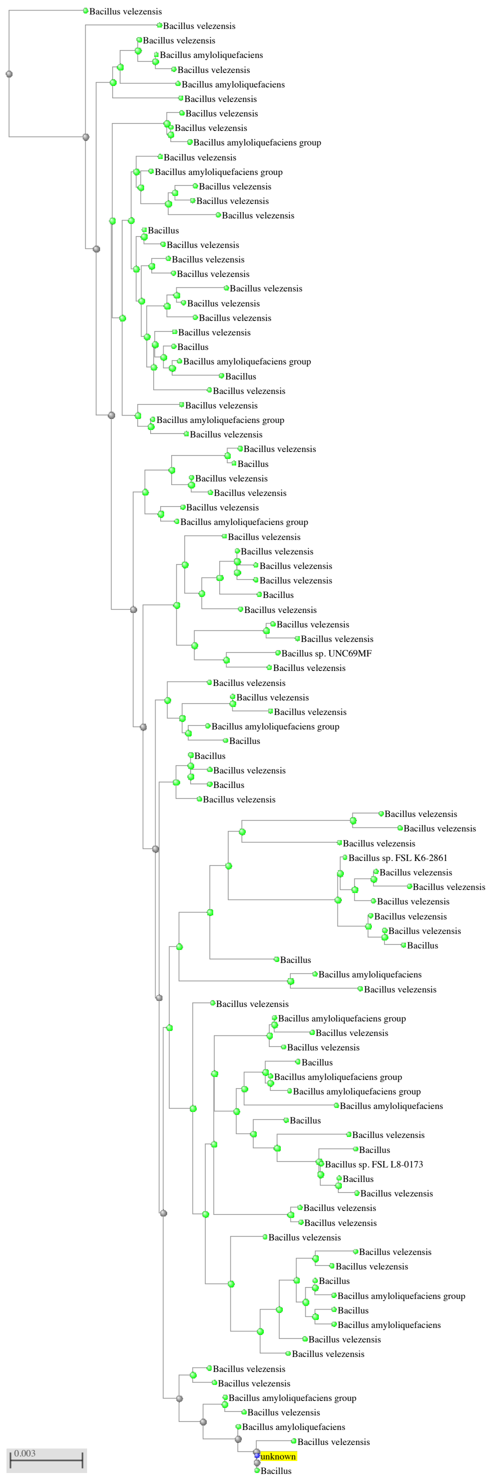

Characterization of the NRPS operon homolog for surfactin synthesis in *Bacillus* spp., Archives of Microbiology,

Kojiro Ito *et al*; Corresponding author: Kenji Yokota, Tokyo University of Agriculture, yokota@nodai.ac.jp

B

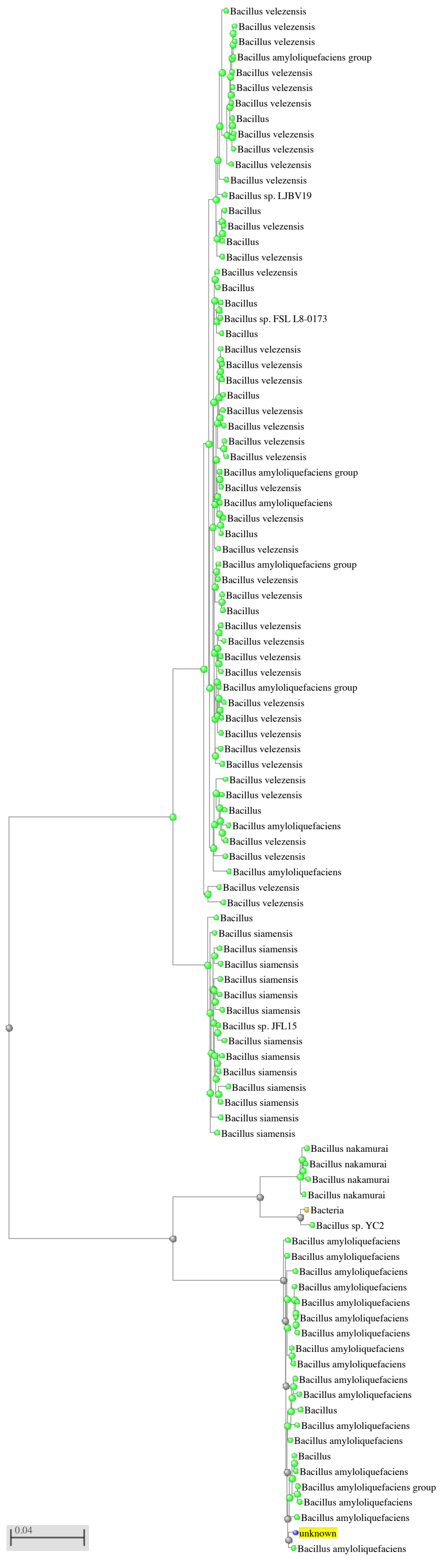

Kojiro Ito *et al*; Corresponding author: Kenji Yokota, Tokyo University of Agriculture, yokota@nodai.ac.jp

Supplementary Fig. S3: BlastP research for *BvelsrfAC* and *BamysrfCC*

A: BlastP analysis of *BvelsrfAC* in *B. velezensis* TUA12

B: BlastP analysis of *BamysrfCC* in *B. amyloliqufaciens* Ptrs2
